# Editorial: Accelerating Genetic Gains in Pulses

**DOI:** 10.3389/fpls.2022.879377

**Published:** 2022-04-07

**Authors:** Aditya Pratap, Shiv Kumar, Patricia L. Polowick, Matthew W. Blair, Michael Baum

**Affiliations:** ^1^Indian Institute of Pulses Research, Indian Council of Agricultural Research, Kanpur, India; ^2^International Center for Agricultural Research in the Dry Areas, New Delhi, India; ^3^National Research Council, Saskatoon, SK, Canada; ^4^Department of Agricultural and Environmental Sciences, Tennessee State University, Nashville, TN, United States; ^5^International Center for Agricultural Research in the Dry Areas, Rabat, Morocco

**Keywords:** pulses, legumes, improvement, plant breeding, QTLs

Legumes, members of the Fabaceae/Leguminosae family, are the third largest family of higher plants with almost 20,000 species belonging to 650 genera, and are ubiquitous all over the world. Among all legumes, pulse crops or food legumes fall into the four clades of the subfamily Papilionoideae which include aeschynomenoids/dalbergiods, genistoids, hologalegina, and phaseoloids/millettoids. They are distinctive due to their positive impact on agricultural and environmental sustainability and have a prominent role in promoting human and animal health, soil amelioration, cropping system diversification, and sustenance of rural livelihoods (Pratap et al., 2021a). These also provide protein isolates that are increasingly being used in the food industry as functional ingredients suitable for vegan diets (Robinson et al., 2019). The inclusion of pulses in rotation with cereals helps to improve system yields, enhance net carbon sequestration, and lower the carbon footprint. Nonetheless, in addition to being an excellent source of protein, starch, and micronutrients, pulses also contain anti-nutritional compounds that can interfere with the absorption of minerals (Moore et al., 2018) and also the digestion of protein (Clemente et al., 2015).

Realizing their importance, significant research has been dedicated to their genetic amelioration, thereby turning them into mainstream crops from so-called “orphan legumes”. Classical plant breeding methods led to the development of more than 3,800 improved varieties of different pulse crops globally, with improved attributes of grain yield, crop duration, stress resistance, nutrition quality, etc. However, despite this effort, the increase in average pulse yields (from 637 to 1,009 kg/ha) has been modest compared to dramatic increases in cereal productivity (from 1,353 to 4,074 kg/ha) between 1961 and 2017 (Kumar et al., 2020). Among legumes, Koester et al. (2014) studied 80 years of historical data of soybean breeding at the Crop Research and Education Center in Urbana, USA and reported a genetic gain of 26.5 kg ha^−1^ year^−1^, attributing the gain in grain yield to increases in light interception, energy conversion, and partitioning efficiencies. Productivity gains in pulses have been recorded when especially considered along with the markedly reduced duration of the improved varieties, leading to increased cropping intensity, while genetic gains have been recorded for traits imparting resistance to major biotic and abiotic stresses, herbicide tolerance, larger seeds, and improved nutritional quality. This resulted in the growth, in terms of production and productivity, in major pulse-producing countries. For example, India witnessed the highest growth in production in mung bean (178%), followed by chickpea (125%), urdbean (90%), pigeonpea (51%), and lentil (34%) in the last 15 years (Gaur, 2021). Notably, breeding in most pulses has remained confined to the exploitation of genetic variation within the primary gene pool, which has resulted in a narrow genetic base in most of them.

Therefore, there is a need to focus on exploiting the genetic and genomic resources made available through draft genome sequences, high-throughput genotyping and phenotyping tools, data management services, and bioinformatics resources. The need to make use of available information in different pulses provided the impetus for this Research Topic.

Globally, chickpea (*Cicer arietinum* L.) is the second largest pulse crop, cultivated by smallholder farmers in more than 50 countries. In recent years, remarkable progress was made in developing novel genomic tools in chickpea including the draft genome sequence (Varshney et al., 2018), millions of single nucleotide polymorphism (SNP) markers (Thudi et al., 2016; Varshney et al., 2019), and cost-effective genotyping platforms including low- to high-density SNP arrays (Roorkiwal et al., 2020). Likewise, quantitative trait loci (QTLs) and markers associated with abiotic and biotic stresses were also identified which facilitated development of superior cultivars through marker-assisted breeding. Thudi et al. reported novel genetic loci associated with root morphological traits, as well as phosphorus-acquisition and use efficiency in chickpea through genome-wide association mapping. They reported an SNP locus (Ca1_12310101) on Ca1 associated with physiological P-use efficiency, shoot dry weight, and shoot P content. They also identified genes related to shoot P concentration, physiological P-use efficiency, specific root length, and manganese concentration in mature leaves. Jha et al. identified major QTLs and potential candidate genes for heat stress tolerance and reported a genomic region on CaLG07 harboring QTLs explaining > 30% of the phenotypic variation for days to pod initiation, 100-seed weight, and for nitrogen balance index explaining > 10% PVE.

The MutMap approach targets discovery of mutant genes for assessing gene function and is based on BSA analysis of mutant progenies obtained in the/an F_2_ population (Etherington et al., 2014). It relies on the cross between the mutant and its wild-type, and thereby directly targets the causal SNPs generated during mutagenesis which are responsible for phenotypic behavior (Tribhuvan et al., 2018). Manchikatla et al. reported development of markers associated with early flowering and enhanced seed size in chickpea through the MutMap approach. They identified a single unique genomic region on Ca6 (between 9.76 and 12.96 Mb) harboring 31, 22, 17, and 32 SNPs with a peak of SNP index = 1 for low bulk for flowering time, high bulk for flowering time, high bulk for 100-seed weight (HSW), and low bulk for HSW, respectively. They developed two markers, viz., Ca6EF10509893 for early flowering and Ca6HSDW10099486 for HSW, and validated them using the candidate SNPs.

Madurapperumage et al. discussed chickpea as a source of essential fatty acids and focused on plant lipids, their functions, and benefits for human health. They reviewed the chemical analysis of essential fatty acids and possible breeding targets to enrich them which could be possible by phenotyping diverse chickpea germplasm; candidate genes responsible for quantitative trait loci mapping using genome-wide association mapping were identified.

Crop wild relatives, landraces, and exotic germplasm are highly useful for introgression of novel variation to widen the genetic base of the elite gene pool leading to incremental gains over the breeding cycles (Pratap et al., 2021a). They also harbor positive QTLs for improving agronomic traits. Toker et al. reported a new *Cicer* species proposed as *Cicer turcicum* and explained its potential to improve various traits in chickpea including heat tolerance and bruchid resistance.

The breeding cycle usually takes 7–10 years for development of a new cultivar depending upon various factors (Kumar et al., 2020). Speed breeding can improve genetic gains in crop improvement programs by increasing the number of plant generations in a year, subsequently reducing the length of the breeding cycle (Chiurugwi et al., 2019). Fang et al. demonstrated a cost-saving speed-breeding methodology in soybean integrating an off-site nursery, a fresh-seeding method, and marker-assisted selection. Using the above combination they could obtain at least four generations in a year against one achieved through conventional methods. Croser et al. demonstrated the effectiveness of collaborative breeding efforts toward deployment of innovative breeding technologies for developing and rapidly introgressing imidazolinone group 2 herbicide tolerance into an Australian *desi* chickpea cultivar. They elaborated that their inter-institutional collaborative efforts could save a period of 3 years.

Root systems have an important role in water and nutrient acquisition, and plant root system architecture (RSA) directly controls the plant health and survival (Sozzani and Iyer-Pascuzzi, 2014). *In situ* methods are available to facilitate non-destructive spatial and temporal investigations into root systems grown in soil. Mung bean [*Vigna radiata* (L.) Wilczek] is a short duration and widely adaptable pulse crop known for its easily digestible high-quality protein (Pratap et al., 2021b), and high initial growth vigor which warrants study for the underlying mechanisms of faster water and nutrient uptake. Singh and Bell reported genotypic variability for RSA in mung bean and their physiological relationships with shoot growth dynamics. Early maturing varieties exhibited rapid root elongation rates and leaf area development. This resulted in more vigorous root and shoot growth during initial crop stages with the early varieties recording deeper, longer, and lighter roots. Rohilla et al. reported 10 marker-trait associations in mung bean significant for yellow mosaic disease, caused by mung bean yellow mosaic India virus, and four seed yield-related traits, viz., days to flowering, days to maturity, plant height, and number of pods per plant. They grouped different genotypes into three major clusters and three genetically distinct sub-populations with one admixture sub-population.

Sadras et al. quantified the genetic gain in lentil yield in the last three decades in Australia and observed the variation in the expression of genetic gain with the environment. They reported that yield did not increase in farmers' fields above the level of 1.2 t ha^−1^ over three decades; this could be attributed to non-mutually exclusive reasons including lack of genetic gain in yield, lack of progress in agronomic practices, lesser adoption of superior technologies, and expansion of lentil to low-yielding environments. Tripathi et al. reported development of a core set of lentil germplasm comprising 170 accessions (137 Indian and 33 exotic) from the Indian gene bank which could be efficiently deployed in lentil improvement programs.

Minor pulses hold tremendous nutritional significance although these are produced and consumed locally and there is generally no exchange of materials between countries unless they are already cultivating and consuming these minor pulses (Ahlawat et al., 2016). Among the minor pulses, faba bean (*Vicia faba* L.) is the fourth most important cool season legume consumed locally in Asia, America, and Mediterranean countries. The genome information is scarce in this crop mainly due to the intrinsic difficulties of assembling/annotating its big genome size of 13 Gb (Maalouf et al., 2021). Adhikari et al. reviewed the development and role of conventional and molecular breeding tools for accelerating genetic gain in faba bean and suggested that the availability and use of DNA markers such as vicine-convicine (*vc*^−^) and herbicide tolerance in breeding programs have encouraged breeders and given confidence in marker-assisted selection. Closely linked QTLs for biotic and abiotic stress tolerance are available and could be used to enhance the efficiency of the selection process. Omomowo and Babalola reviewed the constraints and prospects of improving cowpea productivity to ensure food and nutritional security and environmental sustainability, taking a special interest in Africa. They elaborated on recommended methods to achieve extreme growth in productivity. Chu et al. elucidated the role of *VaSDC1* in modulation of flavonoid metabolic pathways in seed coat color in adzuki bean (*Vigna angularis* L.) and genetically mapped it in the interval between simple-sequence repeat (SSR) markers *Sca326-12, Sca326-4*, and *BAgs007* on chromosome 3 using an F_4_ population.

Of late, there has been a surge in the demand for plant-based proteins globally, even in those countries who have not normally been large consumers of pulses. This warrants a focus on strategies leading to a significant production improvement of pulses and their nutritional quality enhancement. Overall, the 14 articles published in this special issue reported new innovations/contributions toward genetic improvement of pulse crops and the knowledge gained could be further deployed toward development of new superior lines leading to improved genetic gains. The articles also highlight development of new markers, useful marker trait associations, new useful species, etc. Nonetheless, it is also evident that strategically important minor legumes still lag behind other pulses and require a research impetus toward development of new genomic information and deployment of molecular tools for their improvement. Extensive studies are required to quantify precise genetic gains in pulses with respect to yield and nutrition traits which will help in developing strategies for targeted breeding.

## Author Contributions

All authors listed have made a substantial, direct, and intellectual contribution to the work and approved it for publication.

## Conflict of Interest

The authors declare that the research was conducted in the absence of any commercial or financial relationships that could be construed as a potential conflict of interest.

## Publisher's Note

All claims expressed in this article are solely those of the authors and do not necessarily represent those of their affiliated organizations, or those of the publisher, the editors and the reviewers. Any product that may be evaluated in this article, or claim that may be made by its manufacturer, is not guaranteed or endorsed by the publisher.
